# Newly recognized mosquito-associated viruses in mainland China, in the last two decades

**DOI:** 10.1186/1743-422X-8-68

**Published:** 2011-02-14

**Authors:** Hong Liu, Xiaoyan Gao, Guodong Liang

**Affiliations:** 1 State Key Laboratory for Infectious Disease Control and Prevention, Institute for Viral Disease Control and Prevention, Chinese Center for Diseases Control and Prevention, Beijing, China

## Abstract

There are four principal arboviruses in mainland China. Two kinds of them are mosquito-borne viruses, namely Japanese encephalitis virus and dengue virus, which lead to Japanese encephalitis, and dengue fever/dengue hemorrhagic fever respectively; the other two are tick-borne viruses, namely tick-borne encephalitis virus and Crimean-Congo hemorrhagic fever virus (also known as Xinjiang hemorrhagic fever virus), which contribute to tick-borne encephalitis and Xinjiang hemorrhagic fever respectively. With exception of these four main arboviruses, many other mosquito-associated viruses have been isolated and identified in recent years. These newly isolated and identified mosquito-associated viruses are probably responsible for human and animal infections and diseases. The purpose of this review is to describe the newly isolated mosquito-associated viruses in mainland China which belong to five viral families, including their virological properties, phylogenetic relationships, serological evidence, as well as to appeal the public health concentration worldwide.

## Introduction

Arboviruses comprise a group of viruses that reproduce in sensitive blood-sucking arthropods [1]. There are more than 550 species listed in the international catalog, of which more than 128 are known to infect humans and livestock and most are mosquito borne [2]. At present, dengue virus (DENV), Japanese encephalitis virus (JEV), West Nile virus (WNV) and other mosquito-borne viruses are major causes of infectious diseases worldwide [3-6]. Therefore, arboviruses and their related diseases not only are research subjects for virologists but also raise social issues associated directly with public health and attract great public concern [5,6].

Since the 1950 s, four kinds of arbovirus-related diseases have been confirmed to be endemic in China, namely Japanese encephalitis (JE), dengue fever (DEN), tick-borne encephalitis (TBE) and Crimean-Congo hemorrhagic fever (CCHF) (also known as Xinjiang hemorrhagic fever, XHF) [7-10]. The former two kinds of arboviral diseases were caused by mosquito-borne arboviruses: Japanese encephalitis virus (JEV) and dengue virus (DENV), respectively. The latter two kinds of are arboviral diseases were caused by tick-borne arboviruses: tick-borne encephalitis virus (TBEV) and Crimean-Congo hemorrhagic fever virus (CCHFV) (also known as Xinjiang hemorrhagic fever virus, XHFV). JE and DEN are nationally notifiable communicable diseases in China while TBE and XHF are locally reported communicable diseases [7-11]. These four arbovirus infections contribute to a large disease burden in China and have been reported previously and well evaluated [7,12-14]. However, lots of arboviruses have been newly found and identified in mainland China, and the diseases or infections due to these newly isolated arboviruses maybe underestimated.

**Figure 1:**
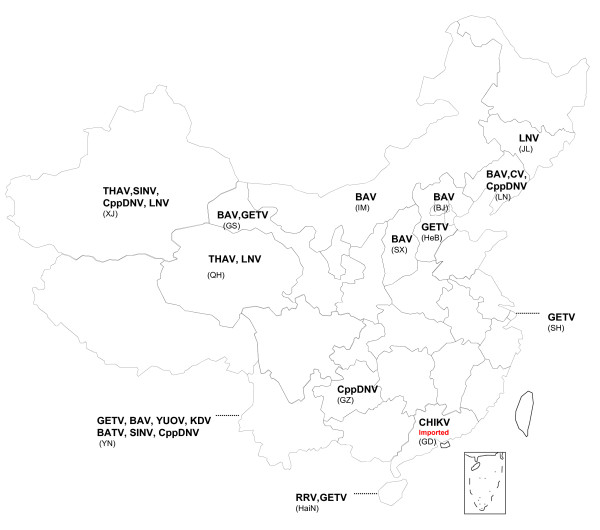
**Distribution of newly isolated mosquito-associated viruses in mainland China**.

This article focuses on the mosquito-associated viruses isolated from variety of arthropods, humans and animals that have been identified in mainland China in recent years, which belong to 5 viral families, and a lot of them are important zoonotic pathogens (Figure 1).

### Bunyaviridae

#### Tahyna virus (TAHV)

**Figure 2:**
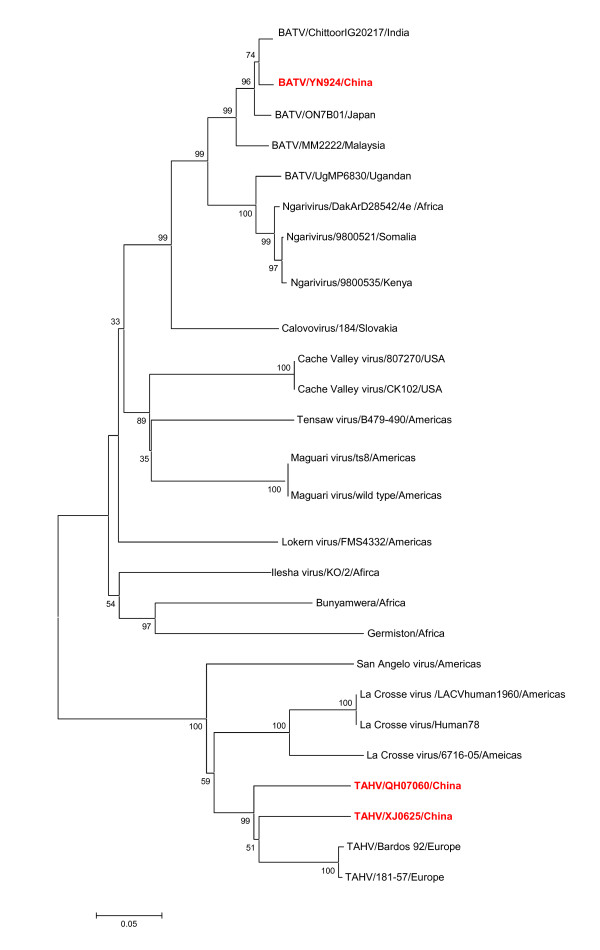
**Phylogenetic analysis based on the nucleotide sequence of the M segment of viruses belonging to the family Bunyaviridae referred to in this review**. Phylogenetic analyses were performed by the neighbor-joining method with MEGA software, version 4 http://www.megasoftware.net/. Bootstrap probabilities of each node were calculated with 1,000 replicates. Scale bars indicate a genetic distance of 0.05-nt substitutions per site. The Chinese isolates are designated by boldface type in red.

A strain of virus (XJ0625) was isolated from *Culex *spp. mosquitoes collected from corrals in Xinjiang, China, in the summer of 2006 by Lu *et al. *[14]. Microplate plaque-reduction neutralization tests were performed using BHK-21 cells and ascites fluid with immunity to prototype TAHV (Bardos 92; provided by the Centers for Disease Control and Prevention, CDC, Fort Collins, CO, USA) to validate its associated cytopathic effects (CPE). These were completely inhibited at ascites fluid dilutions of up to 1:3200. Phylogenetic analysis of XJ0625 from China based on small (S) and medium (M) segments shows that XJ0625 and the prototype of THAV (Bardos 92) are included in the same phylogenetic group (Figure 2). The isolate (XJ0625) was subsequently identified for the first time in China as TAHV belonging to the *Bunyaviridae *family by serological and molecular biology assays (Table 1). Serum samples from patients with fever of unknown cause were collected in the local hospitals. Of the 323 samples, 5.3% (17/323) were IgM positive (detected by indirect fluorescence assay, IFA), and 18% (59/323) were IgG positive. Ten paired serum specimens were assayed by serum dilution neutralization testing with XJ0625 virus on BHK-21 cells. All 10 samples showed neutralizing activity against XJ0625 virus, indicating human infection by TAHV in this region. This was the first report of THAV infection in mainland China [14].

**Table 1:** Mosquito-associated viruses isolated in recent years in mainland China

Virus	strain	Virus isolation				Virus identification						References
				
		Site	Date	Source	Origin	CPE	Sucking mouth death	Virus genome	Antigenicity			
										
									IFA	ELISA	NT	
***Bunyaviridae***												
TAHV	XJ0625	XJ	2006 (Jul)	*Cx. pipiens pallens*	corral	1,2,3	48 h	EU622820	√	/	√	14
	QH07029, QH07060	QH	2007 (Aug)	*Ae. (Och.) detritus*	wheat field	1,2,3	48 h	/	√	/	√	15
BATV	YN92-4	YN	1998 (Jul)	*An. philippinensis*	/	1,2	48 h	/	√	/	√	20
***Flaviviridae***												
CV	Chaoyang virus	LN	2008 (Aug)	*Ae. vexans*	corral	1	/	FJ883471	/	/	/	31
***Togaviridae***												
GETV	M1	HaiN	1964	*Culex mosquito*	/	1,2	48 h	EU015061	√	√	√	32
	HB0234, HB0215-3	HeB	2002 (Sep)	unidentified mosquito	Corral	1,2	/	EU015062	√	/	/	34
	YN0540, YN0542	YN	2005 (Jul)	*Ae. Vexans* *Cx. tritaeniorhynchus*	/	1,2	/	EU015063; EU015064	√	/	/	34
	SH05-6, SH05-15, SH05-16, SH05-17	SH	2005 (Jul)	unidentified mosquito	/	1,2	/	EU015067-EU015070	√	/	/	34
	GS10-2	GS	2006 (Aug)	*Arm. obturbans*	piggery	1,2	/	EU015070	√	/	/	34
												
SINV	YN87448	YN	1987	CSF	/	1,2,3,4,	48 h	AF268026	√	√	√	40
	XJ-160	XJ	1990 (Jul)	*Anopheline mosquito*	wheat field	1,2	48 h	AF103728	/	√	√	41
	MX10	YN	2005 (Aug)	unidentified mosquito	Cow barn	1,2	/	/	√	√	√	/
												
CHIKV	FD08023, FD080178, FD080008, SD08pan	GD	2008 (Mar,Oct, Dec)	Patient	/	3	/	GU199350-GU199353	√	/	√	56
												
RRV	HBb17	HaiN	1993	Bat	/	1,2,5	48 h	/	√	/	√	62
***Reoviridae***												
BAV	BAV Chinese	YN	1987 (Jul)	CSF	/	1	Not dead	AF052030	√	√	/	68
	GS07-KD12, GS07-KD15, GS07-KD16, GS07-KD18, GS07-KD27, GS07-KD29, GS07-KD30, GS07-KD32, GS07-KD38, GS42-2,	GS	2007 (Aug) 2006 (Aug)	*Cx. tritaeniorhynchus* *Cx. pipiens pallens* *An. Sinensis* *Ae. albopictus*	Cow barn piggery	1	Not dead	GQ331954-GQ331962 FJ160414	√	√	/	68
	SX0765, SX0766, SX0767, SX0771, SX0789, SX0790, SX0793, SX0794, SX0795, SX0796	SX	2007 (Aug)	*Cx. pipiens pallens* *Ae. dorsalis* *Ae. vexans*	piggery	1	Not dead	GQ331963-GQ331972	√	√	/	68
	NM0706	IM	2007 (Aug)	*Cx. modestus*	Fish pond	1	Not dead	GQ331973	√	√	/	68
	LN0684, LN0688, LN0689	LN	2006 (Aug)	*An. sinensis*	piggery	1	Not dead	FJ217989-FJ217991	√	√	/	68
	BJ95-75	BJ	1995	Unidentified mosquito	/	1	Not dead	AY568289	√	√	/	68
	YN-6, YN0556, YN0558, YN0659	YN	2001 2005 2006	unidentified mosquito *Cx. tritaeniorhynchus* *An. sinensis*	/	1	Not dead	AY568290, FJ161966; FJ161964, FJ161965	√	√	/	68
												
LNV	LNV-NE9712, LNV-NE9731	JL	1996 (Jul-Sep)	*Ae. dorsalis*	corral	1	Not dead	NC007747, AY710350	√	√	/	73
	0507JS60	XJ	2005	*Culex. mosquito*	corral	1	Not dead	FJ157354	√	√	/	75
	QH07130	QH	2007 (Aug)	*Cx. modestus*	Reed pond	1	Not dead	/	√	/	/	15
												
KDV	YN0559	YN	2005 (Jul)	*Cx. tritaeniorhychus*	corral	1	Not dead	FJ159105	√	√	/	77
												
YUOV	YUOV	YN	1998 (Jul-Aug)	*Cx. tritaeniorhychus*	/	1	Not dead	FJ225402	/	√	/	79
***Parvoviridae***												
CppDNV	JZ-16	LN	2008 Aug	*Cx. pipiens pallens*	corral	1	/	EF579756	√	√	/	80
	YN0569, YN05145, YN05150, YN05152 YN05159, YN05169, YN05217	YN	2005 Aug	*Cx. tritaeniorhychus* *An. sinensis* *Cx. pipiens pallens*	corral	1	/	EF579765-EF579771	√	√	/	80
	XJ0557, XJ0558, XJ0559, XJ0511	XJ	2005 Aug	*Culex. mosqutio* *Cx. pipiens pallens*	corral	1	/	EF579760-EF579764	√	√	/	80
	GZWN1, GZWN2, GZWN3	GZ	2005 Aug	*Culex. mosquito *unidentified mosquito	corral	1	/	EF579757-EF579759	√	√	/	80

Symbols:

"1"represents C6/36 cell; "2"represents BHK-21 cell; "3"represents Vero cell; "4"represents PHK cell; "5"represents Vero-E6 cell

Key

XJ, Xinjiang province; QH, Qinghai province; YN, Yunnan province; HaiN, Hainan province; JL, Jilin province; HeB, Hebei province; SH, Shanghai city; GS, Gansu province; LN, Liaoning province; SX, Shanxi province; BJ, Beijing city; HLJ, Heilongjiang province; IM, Inner Mongolian Autonomous Region; GZ, Guizhou province; IFA, indirect fluorescent antibody test; ELISA, enzyme linked immunosorbent assay; NT, neutralization test; "√" means method used to identify the virus; "/" means information not available.

Two strains of viruses (QH07029 and QH07060) were isolated from *Ochlerotatus detritu *collected in Geermu city located in the Qinghai-Tibet plateau at an altitude of 2800 meters in August 2007, by Li *et al *[15]. The isolates were subsequently identified as TAHV by serological and molecular biology assays (Table 1, Figure 2). A serosurvey demonstrated IgG antibodies against TAHV in 4.4% (16/366) of the residents of Geermu city. In addition, the incidences of TAHV IgG antibody-positive cows and sheep were 16.7% (5/30) and 26.7% (8/30), respectively. This represents the first evidence of TAHV infection in residents and livestock in the Qinghai-Tibet plateau. Moreover, this was also the first time that TAHV had been isolated from *Ochlerotatus detritu*, adding the 13th TAHV-positive mosquito species to the known list [15]. These lines of evidence suggest that TAHV transmission cycles involving mosquitoes and susceptible vertebrate hosts had been maintained in the locality.

TAHV, a member of the California serogroup, genus *Orthobunyavirus*, family *Bunyaviridae*, was first isolated in the former Czechoslovakia in 1958 [2] and is widely distributed in western Asia and central Europe [14-18]. TAHV has been isolated from various natural hosts and vectors such as *Culex pipiens*, *Aedes *sp. mosquitoes and febrile patients [19]. Anti-TAHV antibodies have been observed in serum samples of healthy populations of cattle, sheep, bears, hares, rodents and a variety of birds [17,18]. Human illness arising from infection with TAHV has been reported as manifesting undifferentiated fever and influenza-like symptoms and occasionally pneumonia-like central nervous system involvement [19].

#### Batai virus (BATV)

A strain of virus (YN92-4) was isolated from *Anopheles Philippines *captured in Yunnan province in 1998 by Zhang *et al *[20]. The isolate showed a high response against ascites fluid carrying immune activity against *Bunyavirus *and BATV (Table 1). Sequencing and analysis of a small segment of YN92-4 indicated that it was a BATV [21]. The full coding region of YN92-4 has been sequenced and analyzed by Wang *et al. *[22]. This is the first BATV strain in the world to have had its full coding region sequenced. The phylogenetic analysis of BATV based on S, M and large (L) segments showed that BATV (YN92-4) together with the prototype of BATV (MM2222) and a Japanese isolate (ON-7/B/01) are clustered in one phylogenetic group, further indicating that YN92-4 is a BATV (Figure 2). The reconstructed phylogenetic tree shows that Chinese YN92-4 and Ngari virus (a genetic reassortant virus) belong to two different phylogenetic groups, indicating that no reassortment has occurred in YN92-4 (Figure 2). A serosurvey demonstrated that the BATV specific antibody positive rate was 4.7% (5/120) in febrile patients in Xishuangbanna in Yunnan province where the virus was initially isolated, giving evidence of BATV infection in this locality [23].

BATV was first isolated from *Culex pipiens *in Malaysia in 1955 [2]. Additional BATV isolates have been isolated from mosquitoes, cattle, pigs and febrile patients since then [24-26]. Human infections with BATV show symptoms including headache, fever and occasionally central nervous system involvement [27]. A large hemorrhagic fever (HF) outbreak occurred in 1997-1998 in eastern Africa, causing 89,000 human infections and 250 deaths [28,29]. Analysis showed that the Ngari virus responsible for the HF outbreak is a genetic reassortant virus with S and L segments derived from the Bunyamwera virus and an M segment derived from BATV. This finding attracted great public concern worldwide [30].

### Flaviviridae

#### Chaoyang virus (CV)

**Figure 3:**
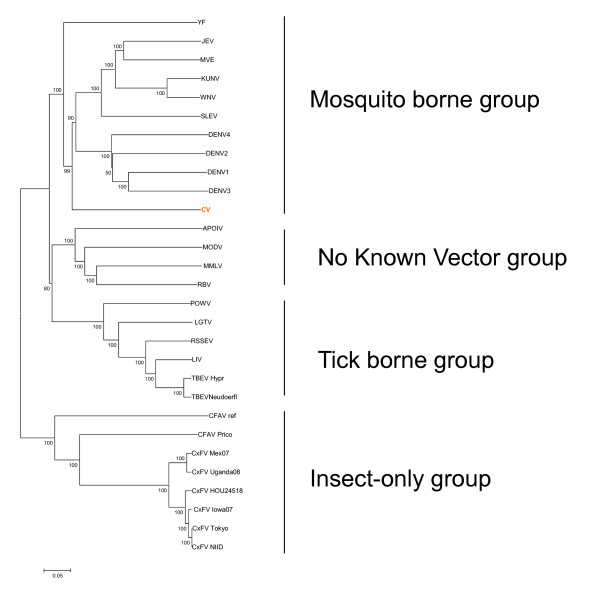
**Phylogenetic analysis based on the full coding sequences of Chaoyang Virus (CV) together with arthropod-borne flaviviruses and flaviviruses with not known vectors**. Phylogenetic analyses were performed by the neighbor-joining method with MEGA software http://www.megasoftware.net/. Bootstrap probabilities of each node were calculated with 1,000 replicates. Scale bars indicate a genetic distance of 0.05-nt substitutions per site. Note that the Chinese CV strains designated by boldface type in red are clustered in the mosquito-borne group and have been separated into an independent evolutionary clade.

This virus was first isolated from *Aedes vexans *in rural corrals in Chaoyang city in Liaoning province in China [31]. CV induces CPE in C6/36 cells leading to cell deformation, disordered arrangement, aggregation and death. The full coding region of CV has been sequenced and analyzed (Table 1). The genome of CV is a positive-sense single-stranded RNA molecule spanning 10308 base pairs with a single open reading frame (ORF) encoding three structural and seven nonstructural proteins. The nucleotide homology of the E gene between CV and the St. Louis encephalitis virus belonging to the JE virus group was the highest at 59.6%; that of the NS3 gene to the Kedougou virus belonging to the DENV group was the highest at 61.7% and that of the *NS5 *gene between CV and the Sepik virus belonging to the yellow fever (YF) group was the highest at 67.0%. The phylogenetic analysis of CV together with other flaviviruses showed that CV clusters in the mosquito-borne group but is located in different phylogenetic branches from other mosquito-borne and tick-borne forms. These findings indicate that CV is a new species of the genus *Flavivirus *within the *Flaviviridae *family [31] (Figure 3). Notably, the phylogenetic relationships of CV with other medically important flaviviruses are close, and *Aedes vexans*, the vector of CV, is widely distributed around the world [2]. Hence, further research on CV is highly recommended to determine its potential virulence, its ability to invade new regions and its potential public and veterinary health problems.

### Togaviridae

#### Getah virus (GETV)

**Figure 4:**
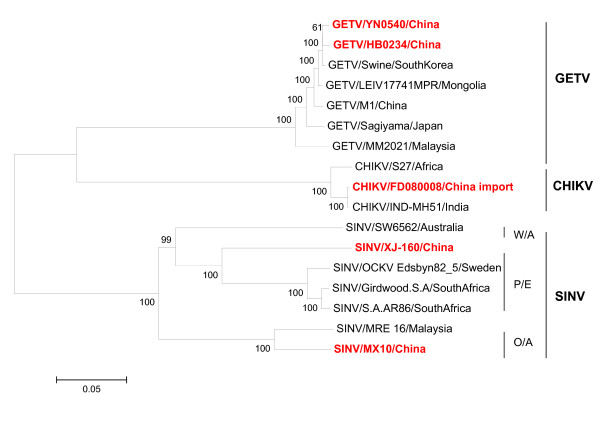
**Phylogenetic analysis based on the full coding sequences of Alphavirus isolated in China together with those referred in this review**. Phylogenetic analyses were performed by the neighbor-joining method with MEGA software http://www.megasoftware.net/. Bootstrap probabilities of each node were calculated with 1,000 replicates. Scale bars indicate a genetic distance of 0.05-nt substitutions per site. The Chinese isolates are all shown in boldface in red.

A strain of virus (M1) was isolated from *Culex *mosquitoes in Hainan province in 1964 by Yang *et al *[32] (Table 1) and was subsequently identified as an isolate of GETV by serological and genomic analyses [33]. Additional GETV isolates have been isolated from mosquitoes collected from Shanghai city and Hebei, Gansu and Yunnan provinces [34,35] (Table 1). Ten strains of GETV have been isolated from different mosquito species including *Aedes vexans*, *Armigeres obturbans*, *Culex *spp. and unidentified mosquitoes from China, representing a wide geographic distribution and a long time interval. These have been sequenced and analyzed [35]. Full-length genome sequences were determined for three isolates: M1, HB0234 and YN0540. The remaining seven isolates have had the E2 gene and the 3' untranslated region (UTR) sequenced. The nucleotide (nt) homology of the E2 gene between these 10 strains of virus is 97.7-99.9%. The nucleotide sequence identity of the full-length genome sequences between the three Chinese isolates ranges from 98.1% to 99.1%, while the amino acid (aa) sequence identity ranges from 98.7% to 99.8%. The Chinese, Russian and Mongolian isolates show the same deletion (10 nt) at positions 45-54 in the 3' UTR, making it a molecular marker for the GETV isolates in these regions [35] (Figure 4).

GETV was first isolated from *Culex gelidusin *in Malaysia in 1955 [2]. The virus is known as a pathogen of horses and pigs [36,37]. Neutralizing antibodies to GETV have been identified in serum samples from humans and birds in Malaysia, northern Australia and Hainan province in China [32,38,39]. Despite these findings, the virus has not been linked to illness in humans.

#### Sindbis virus (SINV)

Three new strains of this virus were isolated in China in 1987 (YN87448) [40], 1990 (XJ-160) [41] and 2005 (MX10) [Wang JL, Zhang HL, Sun XH, Fu SH, Wang HY, Wang HQ, Liang GD: Distribution of mosquitoes and mosquito-borne arboviruses in Yunnan Province near the China-Myanmar-Laos border, Submitted], respectively. SINV could be divided into three genotypes: Paleoarctic/Ethiopian (P/E), Oriental/Australian (O/A) and Western/Australian (W/A) [42]. Phylogenetic analysis suggested that YN87448 and XJ-160 belong to the P/E genotype; however, XJ-160 virus was clustered in a separate clade and must have evolved independently [41]. The nucleotide homology between MX10 and Malaysia isolate MRE-16 was 90.0%, and MX10 belongs to the O/A genotype (Figure 4).

Since the isolation of XJ-160 in 1990, studies on XJ-160 have been conducted including whole genome sequence [41], full-length infectious cDNA cloning [43]. Based on the full-length cDNA clone of XJ-160, the effects of aa substitutions in the nonstructural proteins nsP1 and nsP2 on the infectivity and pathogenesis of the virus have been investigated [44,45]. Packaging cell lines (PCLs) of the XJ-160 virus have been constructed to develop an XJ-160 virus-based vector system [46]. In addition, the essential role of the E2 glycoprotein has been confirmed in XJ-160 viral infections, especially the 145-150 aa domain acting through interaction with cellular heparan sulfate [47].

A serosurvey has demonstrated SINV-specific antibodies in humans and animals in several areas in China [48]. Antibodies to SINV have been identified in healthy individuals as well as febrile patients, and in dogs and voles in Yunnan, Hainan and Guangdong provinces [49,50]. In Fujian province, the seropositive rate in the healthy individuals was 12.9% (27/210), and SINV IgG antibodies could be detected in foxes, rabbits and dogs [51]. In addition, IgG antibodies to SINV were detected from patients with unknown fever and encephalitis and leptospiral meningitis [52], indicating that SINV might be a pathogen causing fever and viral encephalitis in China, so enhanced surveillance of SINV and its infection are in dire need.

SINV was first isolated from a pool of *Culex *sp. mosquitoes in 1952 in the Nile river delta in Egypt [2]. The virus was widely distributed in the world [53]. The primary hosts and vectors are birds and mosquitoes, and large animals and humans act as definitive hosts [54]. SINV infection is called "Sindbis disease" and is clinically manifested by rash and arthritis; therefore, SINV infections are of great public health importance [55].

#### Chikungunya virus (CHIKV)

In 2008, five cases of CHIKV imported from Malaysia were detected in Guangzhou city. All were confirmed by laboratory testing, and four strains of CHIKV were recovered from the samples [56] (Figure 4). This evidence indicated that the introduction and spread of CHIKV outbreaks in China is a potential threat. Recently, a CHIKV outbreak occurred in Dongguan city, Guangdong province in October, 2010. The lab testing showed that 10 out of 15 sera samples were confirmed to be CHIKV nucleotide positive. Also about 91 suspected CHIKV infection cases were tracked through epidemiological investigation till 1th, October, 2010[57]. So long-term surveillance and clinical attention are still important.

CHIKV was first isolated from the blood of a febrile patient in Tanzania in 1953 [2]. CHIKV infection can cause an acute debilitating illness, most often characterized by fever, severe joint pain and rash [58]. CHIKV has become the most prevalent *Alphavirus *infection in the world, particularly prevalent in Africa and Southeast Asia [59,60]. In 2005, there was an epidemic in India and in countries around the southwest Indian Ocean [61].

#### Ross River virus (RRV)

A strain of this virus (HBb17) was isolated from brain tissue of bat in Hainan province in China by Zhao *et al. *[62] (Table 1). Immunofluorescence assays and cross-neutralization testing demonstrated that the strain has a close relationship with RRV. Molecular analysis of RRV showed that the nucleotide homology of the 3' UTR and *E1 *gene between HBb17 and the RRV prototype (T48) was 99.0%. Phylogenetic tree analysis showed that the HBb17 strain was in the same phylogenetic branch as RRV [63] (Figure 4). In experimental conditions, the isolate HBb17 could be replicated in mosquitoes and caused mice to be infected and to die following bites from infected mosquitoes [62].

RRV-specific IgG antibodies were detected in serum samples of healthy individuals and rats in Hainan province by IFA. The positive rates were 1% (1/98) and 8% (6/75) respectively, suggesting that RRV infections exist in Hainan province [62].

RRV was first isolated from *Aedes vigilax *captured in the Australian Ross River region in 1959 [2]. This virus is mainly distributed in south Pacific regions such as Australia and Fiji. RRV infection mainly manifests as fever, rash and polyarthritis [64,65].

### Reoviridae

#### Banna virus (BAV)

BAV was first isolated from patients with unknown fever and encephalitis in Xishuangbanna in Yunnan province located in southern China, 1987 [66] (Table 1). BAV is the prototype species of genus *Seadornavirus *within the family *Reoviridae *[67]. The BAV genome consists of 12 segments of double-stranded RNA (dsRNA), termed segments 1 to segment 12 in order of decreasing molecular mass determined by polyacrylamide gel electrophoresis (PAGE) and agarose gel electrophoresis. The PAGE profile of BAV is a 6-6 pattern [66]. BAV isolates have been obtained from mosquitoes including three genera and 10 species in various provinces of China (Gansu, Shanxi, Liaoning, Yunnan and Beijing) [68] (Table 1) and from mosquitoes in Indonesia and Vietnam [69,70]. Phylogenetic analysis of BAVs isolated from southeast Asia (including Yunnan province in China, Vietnam and Indonesia) based on segment 12 indicated that the BAV isolates could be divided into two phylogenetically different groups according to their geographic origin: south and north [68].

A large-scale serosurvey has been conducted in several provinces in China. Serum specimens of 1141 patients who supposedly had JE or viral encephalitis were tested for anti-BAV IgM antibodies by enzyme linked immunosorbent assay (ELISA); the positive rate was 11.4% (130/1141) [71]. Among the 63 serum specimens of patients diagnosed clinically with viral encephalitis, 11 were anti-BAV IgM antibody positive, 37 specimens were anti-JEV IgM antibody positive and 7 specimens were detected as having JEV and BAV co-infections [72]. Notably, all of the data reported on BAV infections were only tested by indirect ELISA method. And no four-fold or greater rise in BAV specific antibody by neutralization test in serum collected during the acute and convalescent phase of illness was available. In addition, except the first isolate of BAV from patients, no secondary BAV has been isolated from patient again. So it is important to conduct further investigation on BAV, especially the association between BAV and human diseases.

#### Liaoning virus (LNV)

**Figure 5:**
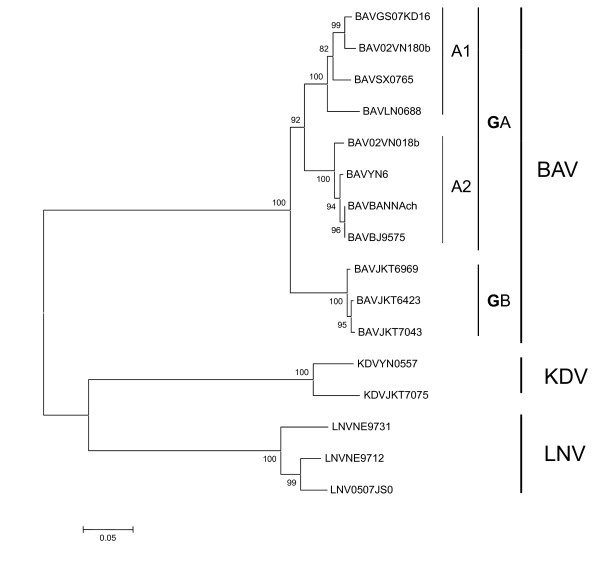
**Neighbour-joining phylogenetic tree built from the segment 12 sequences of different members within the Seadornaviruses**. Phylogenetic analyses were performed by the neighbor-joining method with MEGA software http://www.megasoftware.net/. Bootstrap probabilities of each node were calculated with 1,000 replicates. Scale bars indicate a genetic distance of 0.05-nt substitutions per site.

This virus (LNV-NE9712) was first isolated from *Aedes dorsalis *collected in Heishan in Jilin province in China in 1997 [73]. LNV is a member of the genus *Seadornavirus *within the family *Reoviridae *and is composed of 12 segments of dsRNA [74]. The PAGE profile of LNV is 6-5-1 which is different from that of BAV [75]. In 2005, a strain of virus (0507JSS60) was isolated from corrals in Kashi in Xinjiang province and was identified as LNV by serological and molecular biology assays [75] (Figure 5). In 2007, a strain of virus (QH07130) was isolated from *Culex modestus *collected in reed ponds in Minhe county in Qinghai province [15]. The PAGE profile of QH07130 strain is a typical 6-5-1 pattern. Together with the results of serological and molecular biology detections, QH07130 strain was identified as LNV [15]. LNV could propagate in various mammalian cell lines and caused hemorrhaging in mice, indicating the potential threat to health of human and livestock [76]. However, there has been no report that LNV is associated with human or livestock diseases. Further study of LNV especially on its virulence is highly recommended.

#### Kadipiro virus (KDV)

KDV was first isolated from mosquitoes collected in Indonesia [69]. KDV belongs to the genus *Seadornavirus *within the family *Reoviridae *[74]. Five strains of this virus were isolated from *Culex tritaeniorhynchus*, *Anopheles sinensis *and *Armigeres obturbans *in Yunnan province in China in 2005. A 758 bp amplicon was obtained using KDV-specific polymerase chain reaction primers for the five strains [77]. The nucleotide homology between the KDV Chinese isolates and KDV prototype (JKT-7075) was 90%, indicating that these five isolates are indeed KDV (Figure 5). This was the first report of KDV being isolated outside Indonesia [78]. At present, there is no report that KDV is associated with human or livestock diseases.

#### Yunnan Orbivirus (YUOV)

YUOV is a newly identified member of genus *Orbivirus *within the family *Reoviridae *[79]. It was first isolated from *Culex tritaeniorhynchus *in Lanchang County in Yunnan province in southern China. Electron micrography of YUOV particles showed a defined surface structure, with ring-shaped capsomeres that are characteristic of *Orbivirus *core particles [79]. The viral genome is composed of 10 segments with conserved terminal sequences similar to those of other *Orbivirus *species. The agarose gel electrophoretic profile shows a 3-2-5 pattern. YUOV could replicate in *Aedes albopictus *mosquito cell lines C6/36 and AA23 but could not replicate in mammalian cells. Intraperitoneal injections of YUOV into mice resulted in productive, nonlethal virus replication and viremia, and could induce production of a protective antibody. The evolutionary relationship of YUOV to 11 other species of the genus *Orbivirus *shows that it is a newly identified species [79]. In recent years, six strains of *Orbivirus *have been isolated from *Culex tritaeniorhynchus*, *Anopheles sinensis*, *Culex fuscocephala *and *Anopheles philippinensis*. Serosurveys demonstrated prevalence rates of 0.7% (1/135) of YUOV IgM antibodies and 5.9% (8/135) of IgG antibodies in febrile patients in Yunnan province, giving evidence of YUOV infection in the area [Wang JL, Zhang HL, Sun XH, Fu SH, Wang HY, Wang HQ, Liang GD: Distribution of mosquitoes and mosquito-borne arboviruses in Yunnan Province near the China-Myanmar-Laos border, Submitted]

### Parvoviridae

#### Culex pipiens pallens densovirus (CppDNV)

During an investigation of arboviruses in China, a new kind of virus with the same biological characteristics and a genome sequence very similar to the viruses belonging to the genus *Brevidensoviruses *within the family *Parvoviridae *was firstly isolated from *Culex pipiens pallens *in Liaoning province. Additional strains of CppDNV were subsequently isolated from various provinces of China (Table 1). The virus was designated CppDNV after the vector from which it was isolated. The virus was subsequently isolated from various kinds of mosquitoes. Analyses of the phylogenetic relationships and the genomic nucleotide sequences of 16 strains isolated from *Culex pipiens pallens *(two strains), *Culex pipiens quinquefasciatus *(two strains), *Culex tritaeniorhynchus *(three strains), *Anopheles sinensis *(one strain), unclassified *Culex *mosquitoes (six strains) and other miscellaneous mosquitoes (two strains) from the Liaoning, Yunnan, Xinjiang and Guizhou provinces in China demonstrated a nucleotide homology of more than 98%. This confirmed that the virus was a newly identified member of the species *Aedes aegypti densovirus *(AaeDNV) along with the previously identified *Aedes albopictus densovirus *(AalDNV-2) [80].

## Conclusions

In the last two decades, 12 species of viruses belonging to five families have been newly identified in mainland China. Four (TAHV, BATV, SINV and RRV) are pathogenic to humans and livestock. Four (BAV, LNV, YUOV and CV) are initially isolated and identified in China and are considered to be potential threats to humans and livestock. One (CHIKV) is an imported virus, and no autochthonous cases have been reported in mainland China to date. One (GETV) is known as a pathogen in horses and pigs. Two (KDV and CppDNV) are associated with insects and are not pathogenic to humans. These species of viruses have been isolated from ten mosquito species in four genera, including *Culex pipiens pallens*, *Culex modestus, Culex tritaeniorhynchus, Aedes (Och.) detritus, Aedes vexans, Aedes dorsalis, Aedes albopictus, Anopheles philippinensis, Anopheles sinensis and Armigeres obturbans*. Of this growing list of arboviruses, most are mosquito borne. The ability of such viruses to spread between geographic regions through movements of people, animals and goods means that addressing them clinically is of great public health importance. Given the increasingly frequent international exchanges with China, the risk for further introduction and exportation of the mosquito-borne viruses via human activity and transportation of goods becomes greater, so enhanced monitoring and long-term surveillance of these viruses are of great public health importance both in China and internationally.

## Competing interests

The authors declare that they have no competing interests.

## Authors' contributions

HL and XYG contributed equally to the analysis and interpretation of the results, and involved in drafting the manuscript. Both of them have read and approved the final version of the manuscript. Dr. GDL contributed to conception and design of the manuscript, and involved in drafting and revising the manuscript as well as gave final approval of the version to be published.
